# Circular Valorization of Yellowfin Tuna (*Thunnus albacares*) Bone By-Products into Bioactive Hydrolysates Using Endogenous Visceral Proteases Compared with Commercial Trypsin

**DOI:** 10.3390/molecules31172996

**Published:** 2026-08-27

**Authors:** Belén Encalada, Alisson Sisa, Karla Garcés, Caterine Donoso, Eugenia Peñaherrera Wilches, Oscar Martínez-Álvarez, Jenny Ruales, Mauricio Mosquera

**Affiliations:** 1 Department of Food Science and Biotechnology, Escuela Politécnica Nacional, Quito 170525, Ecuador; anabelen.encalada@epn.edu.ec (B.E.); alisson.sisa@epn.edu.ec (A.S.); karla.garces@epn.edu.ec (K.G.); caterine.donoso@epn.edu.ec (C.D.); jenny.ruales@epn.edu.ec (J.R.); 2 Departamento de Ciencias de la Energía y Mecánica, Universidad de las Fuerzas Armadas ESPE, Sangolquí 171103, Ecuador; 3 Department of Biosciences, Group of Medicinal Plants and Natural Products, Faculty of Chemistry, Universidad de Cuenca, Cuenca 010202, Ecuador; eugenia.penaherrera@ucuenca.edu.ec; 4 Institute of Food Science, Technology and Nutrition (ICTAN-CSIC), 6 José Antonio Novais St., 28040 Madrid, Spain; oscar.martinez@ictan.csic.es

**Keywords:** bioactive peptides, fish by-products, endogenous proteases, functional ingredients, Artemia salina, ACE inhibition

## Abstract

Yellowfin tuna (*Thunnus albacares*) processing by-products are underutilized resources rich in high-quality proteins that can be valorized into bioactive ingredients. In this study, proteins from yellowfin tuna tail bones were hydrolyzed using either an endogenous protease extract recovered from tuna viscera or commercial trypsin. The endogenous extract was characterized and exhibited optimal proteolytic activity at pH 8 and 40 °C. Comparative bioactivity assessment showed that trypsin-derived hydrolysates exhibited significantly higher antioxidant capacity, with ABTS and DPPH values of 11.8 ± 0.04 and 13.3 ± 0.40 mg Trolox/g, respectively, compared with 10.1 ± 0.12 and 7.3 ± 0.21 mg Trolox/g for hydrolysates produced with endogenous enzymes. Trypsin hydrolysates also showed stronger dipeptidyl peptidase-IV (DPP-IV) inhibition (IC_50_ = 0.83 ± 0.37 mg/mL) than endogenous-enzyme hydrolysates (IC_50_ = 1.60 ± 0.47 mg/mL). Remarkably, hydrolysates generated with the endogenous enzymatic consortium exhibited exceptionally potent angiotensin-converting enzyme (ACE) inhibitory activity (IC_50_ = 0.008 ± 0.004 mg/mL), outperforming trypsin hydrolysates (IC_50_ = 0.01 ± 0.007 mg/mL). Neither hydrolysate showed evidence of acute toxicity in the *Artemia salina* model at the tested concentrations, supporting their favorable performance in this preliminary toxicity screening assay. Considering that nearly two-thirds of total fish biomass is discarded during processing, this integrated bioprocess demonstrates a sustainable strategy for the simultaneous valorization of skeletal and visceral waste streams. By employing endogenous enzymes as biocatalysts, this approach reduces dependence on commercial proteases, mitigates environmental burdens, and supports the development of a circular marine bioeconomy through the production of highly potent, peptide-rich functional ingredients with promising cardiovascular health applications. Further toxicological evaluation is required to confirm their safety for food and nutraceutical applications.

## 1. Introduction

The fishing industry has experienced sustained growth in global demand, reflected by the consumption of 164.6 million tons in 2022 [1], of which approximately 55% corresponds to marine capture fisheries [1]. Within this context, tropical tuna stands out as one of the most economically valuable marine resources, with catches from the Indian Ocean accounting for 21% of global production and generating an estimated value of USD 6.5 billion [2]. The proportion of waste generated (30–75% of the total weight of fish landed) is also substantial [3]. This number highlights the urgent need for innovative strategies to valorize this biomass and mitigate the environmental impacts of its disposal. When inadequately managed, fish processing by-products can rapidly decompose because of their high moisture and organic matter content, generating unpleasant odors, increasing biological oxygen demand in receiving waters, and contributing to greenhouse gas emissions. In addition, their disposal represents a significant economic burden for the seafood industry due to handling, transportation, and waste treatment costs [3].

The recovery of fish by-products has emerged as a promising approach to enhance resource efficiency and promote circular bioeconomy principles. Despite being classified as by-products, these materials remain poorly exploited owing to technological constraints and a lack of research [3,4,5] as well as a negative perception of their utilization [6]. While fish bones, for example, are typically processed into fishmeal for animal feed [7], they represent an excellent source of collagen and gelatin, which, through enzymatic or thermal hydrolysis, can yield bioactive peptides with antioxidant, antihypertensive, and anti-inflammatory properties [8,9]. According to the principles of the circular economy and the bioeconomy, fish processing by-products should be regarded as secondary raw materials rather than waste because they retain valuable proteins, minerals, lipids, and other biomolecules that can be reintroduced into the value chain through biorefinery approaches. This concept is aligned with international strategies promoting sustainable resource use, including the European Green Deal, the Circular Economy Action Plan, and the FAO Blue Transformation initiative, which encourage the valorization of aquatic biomass to reduce waste generation and increase resource efficiency. Within this framework, the conversion of fish bones into high-value bioactive ingredients represents an opportunity to simultaneously reduce environmental impacts and generate new economic value from underutilized marine resources [1].

The most important digestive enzymes recovered from these organs comprise both acidic gastric enzymes, such as pepsin (EC 3.4.23), and alkaline intestinal enzymes, primarily trypsin and chymotrypsin belonging to the serine protease family (EC 3.4.21) [10]. These proteases exhibit high biological activity and have the potential for use in pharmaceutical applications [11], whereas other by-products, such as residual muscle protein, exhibit significant potential as a source of bioactive peptides.

Among the most commercially significant species, Yellowfin tuna (*Thunnus albacares*), commonly known as albacore, rabil, or yellowfin, is the second most captured tuna species worldwide, primarily by industrial fleets [12]. It inhabits tropical and subtropical waters of all oceans except the Mediterranean Sea [13]. This species exhibits remarkable physiological resistance, high metabolic activity, and adaptability to low-nutrient environments, which require a diverse and efficient enzymatic system to sustain rapid growth and continuous protein turnover under fluctuating environmental conditions [14]. As a result, its viscera and digestive tissues are particularly rich in endogenous proteases, primarily trypsin-like enzymes [15]. As endopeptidases, these serine proteases specifically cleave peptide bonds at the carboxyl side of arginine and lysine residues [16]. Notably, marine trypsins often demonstrate higher catalytic efficiency in native protein degradation than their mammalian counterparts (e.g., bovine trypsin), alongside broad substrate specificity and stability across a wide range of pH and temperature values [15,16].

One of the main challenges in producing protein hydrolysates lies in the high cost of commercial enzymes, which accounts for up to 70% of the total process expenses (e.g., Alcalase^®^ or porcine trypsin) [17]. Proteases isolated from tuna viscera offer a cost-effective and sustainable alternative to these commercial catalysts [18]. Furthermore, the use of marine-derived enzymes provides a significant advantage for specialized markets, as they are culturally compatible with Halal and Kosher dietary requirements, avoiding the restrictions associated with porcine-origin enzymes [19,20]. The enzymatic hydrolysis not only releases bioactive peptides but also enhances the techno functionality of the proteins, increasing solubility, foaming and emulsifying capacity, and reducing allergenicity, which amplifies their use in functional foods [21].

Marine protein hydrolysates may exhibit pleiotropic bioactivity due to their capacity to modulate specific physiological processes, primarily through the inhibition of key enzymes linked to chronic pathologies. Some protein hydrolysates have demonstrated significant antihypertensive potential through the inhibition of the Angiotensin-Converting Enzyme (ACE) [22,23]. Furthermore, protein hydrolysates can play a relevant role in glycemic control by acting as inhibitors of dipeptidyl peptidase-IV (DPP-IV) and α-glucosidase [24]. The inhibition of DPP-IV prevents the rapid degradation of incretin hormones, such as GLP-1, thereby enhancing insulin secretion and glucose homeostasis [25]. Simultaneously, the inhibition of α-glucosidase delays the hydrolysis of complex carbohydrates in the small intestine, reducing postprandial glucose absorption [26]. These combined inhibitory effects, alongside their antioxidant and anticancer properties among others, position marine peptides as promising candidates for the development of functional ingredients [27]. These biological activities make marine protein hydrolysates attractive for the rapidly expanding functional food and nutraceutical sectors, which are increasingly driven by consumer demand for naturally derived bioactive ingredients with scientifically supported health benefits. Protein hydrolysates enriched in bioactive peptides are currently being explored as ingredients in functional beverages, dietary supplements, and specialized nutrition products aimed at supporting cardiovascular and metabolic health. Therefore, the development of sustainable processes for producing marine bioactive peptides addresses both environmental sustainability goals and the growing market demand for high-value functional ingredients [21].

To the best of our knowledge, this is the first study to integrate the recovery of endogenous digestive enzymes from yellowfin tuna viscera with the hydrolysis of skeletal by-products from the same processing chain. The proposed approach establishes a closed-loop valorization strategy in which one processing by-product (viscera) is used as the enzymatic source to convert another by-product (tail bones) into bioactive hydrolysates. By simultaneously valorizing two major processing by-products, this integrated bioprocess reduces dependence on commercial enzymes while increasing the added value of materials traditionally regarded as waste. Therefore, this work represents a novel contribution to sustainable marine biorefinery and circular bioeconomy strategies.

The objective of this study was to develop an integrated bioprocess for the simultaneous valorization of visceral and skeletal by-products from Yellowfin tuna. To achieve this, endogenous proteases recovered from tuna viscera were evaluated as sustainable alternatives to commercial enzymes for the production of bioactive hydrolysates from tail bones, with emphasis on their antioxidant, antihypertensive, antidiabetic, and safety-related properties.

## 2. Results and Discussion

### 2.1. Nutritional Characterization of Tail Bone By-Products from Yellowfin Tuna (Thunnus albacares)

The elemental composition of the dried flour showed a protein content of 59.38 ± 0.78% (*w*/*w*). Ash content was 26.43 ± 1.06%, moisture 10.20 ± 0.27%, and lipid content 2.83 ± 0.72%. Several authors have reported protein contents ranging from 27% to 32% after drying and defatting fish bones, as in the case of tuna bone powder, which contains 30.28% protein and 62.80% ash [28]. In comparison, the bones analyzed in this study, with a protein content exceeding 50% [29], represent an ideal raw material for the production of enzymatic hydrolysates.

Regarding lipid and moisture content, it has been determined that fish bones contain less than 4% lipids, a value characteristic of osseous structures, in contrast to muscle tissues, where fat content can exceed 10% [30]. This low lipid level, together with controlled moisture, is essential for preserving the structural stability of proteins and preventing their oxidation or premature degradation during enzymatic hydrolysis processes [31]. In this regard, a study conducted on cod demonstrated that using raw material with moisture levels below 16% promotes the production of more efficient hydrolysates, as lower moisture helps maintain protein integrity and enhances enzymatic activity during the reaction [32].

Given that the bone raw material contained high ash content (26.43%), the resulting hydrolysates are likely rich in essential minerals, particularly calcium and phosphorus [28]. Fish bones are recognized as biomineralized structures, which consist primarily of calcium phosphates, with hydroxyapatite as the predominant crystalline phase [33]. Therefore, utilizing the mineral-dense tuna bone fraction as a source for fortified foods transforms a neglected waste material into a high-value functional ingredient rich in bioavailable minerals.

### 2.2. Proteolytic Activity of the Endogenous Enzyme

An initial screening of the visceral extract exhibited a baseline protease activity of 57.4 ± 0.06 U/mL when casein was used as the substrate, indicating an adequate proteolytic capacity for application in protein hydrolysis processes. The use of casein allows for the precise quantification of released soluble peptides and amino acids (such as tyrosine), providing a reliable estimation of the functional activity of the enzymatic extract [34]. This level of activity suggests that the endogenous enzymes present in the viscera maintain their stability and catalytic efficiency under the tested conditions (37 °C). The measured activity is consistent with requirements for the efficient degradation of complex protein matrices, such as bone tissue, ensuring a sufficient release of bioactive peptides during the subsequent hydrolysis stages. For the hydrolysis experiments, the enzyme preparation was purified, and its proteolytic activity was re-determined. Based on the activity of this purified preparation, an enzyme dosage of 650 U/g of substrate was achieved by adding 10 µL per gram of substrate. Details of the purification procedure and enzyme activity determination are provided in the Materials and Methods section.

### 2.3. Molecular Characterization

#### 2.3.1. Determination of the Molecular Weight Profile of the Enzymatic Visceral Extract (SDS-PAGE)

**Figure 1:**
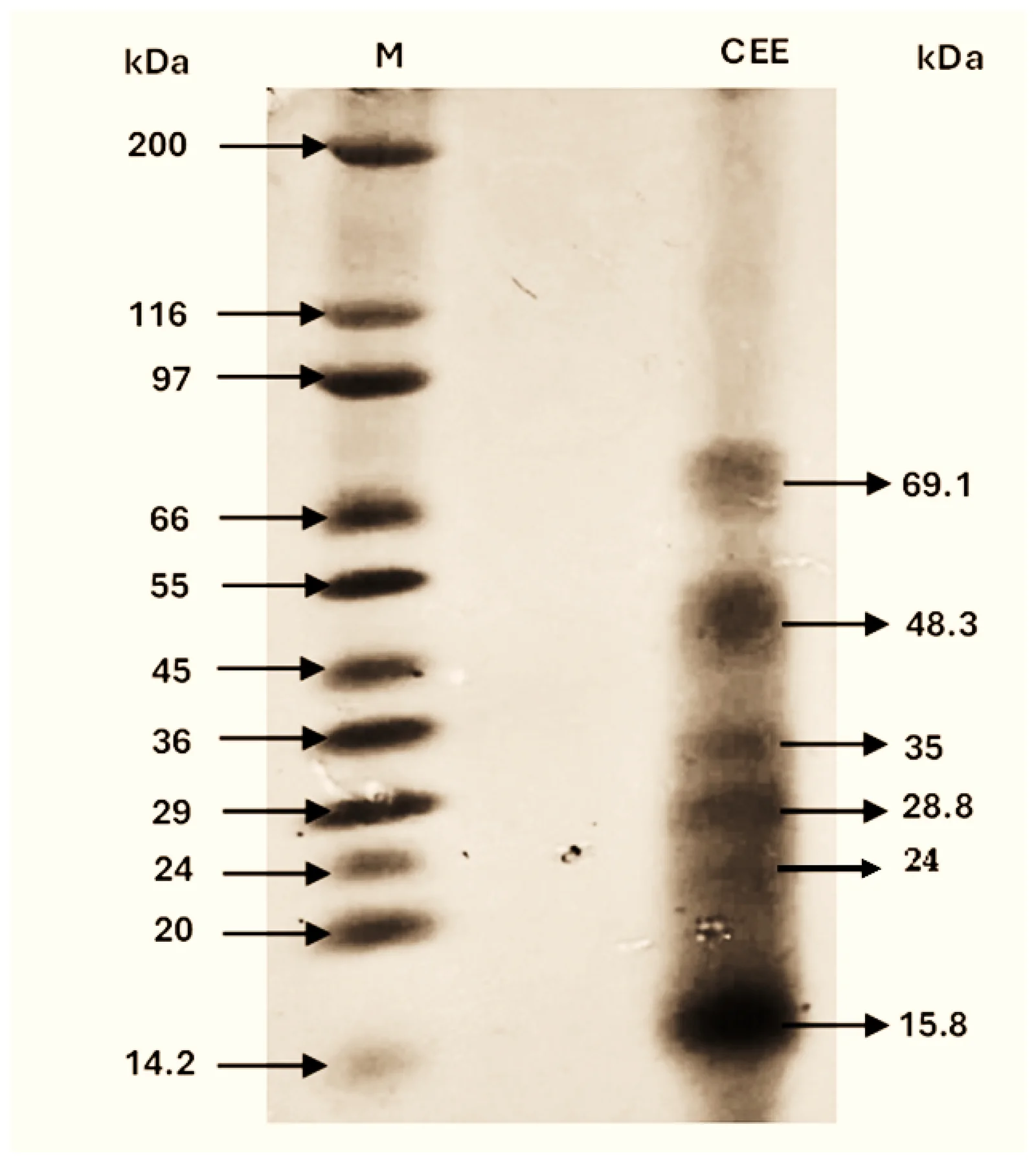
SDS-PAGE of the crude enzymatic extract. Lanes: M = molecular weight marker; CEE = crude enzymatic extract. (Polyacrylamide gel electrophoresis analysis using 10% acrylamide gels prepared from a 49.5%T, 3%C acrylamide/bisacrylamide mixture (T denotes the total percentage concentration of both monomers (acrylamide and bisacrylamide) and C denotes the percentage concentration of the cross-linking agent relative to the total concentration of T)).

Examination of the electrophoretic profile of the crude enzymatic extract (CEE) obtained from yellowfin tuna visceral waste reveals a notably complex band distribution (see Figure 1). This diversity of molecular masses is characteristic of visceral matrices, where the primary endogenous digestive enzymes of the fish gastrointestinal tract inherently coexist with tissue structural proteins and plasma components.

In total, six well-defined protein bands were resolved within a range of 15 to 70 kDa. Based on their relative mobility (Rf) against the standard molecular weight marker, the obtained values corresponded to 69.1, 48.3, 35.0, 28.8, 24.0, and 15.8 kDa. To infer the potential identity of the components in this mixture, these weights were initially contrasted against the BRENDA enzyme database and specialized literature.

This associative analysis suggested that the 28.8 kDa band is consistent with the molecular weight reported for fish trypsin-like proteases, specifically β-trypsin (classified under EC 3.4.21.4). This correspondence precisely aligns with characterization studies for this species, in which purified trypsin from yellowfin tuna intestines exhibited a mass of 29.192 kDa [35], furthermore falling within the general 22 to 30 kDa range established for trypsins derived from marine by-products [10].

On the other hand, the band detected at 24.0 kDa falls within the molecular weight range reported for fish chymotrypsins (22–30 kDa) [10] and pancreatic elastases (25–27 kDa) [36], which frequently co-migrate and overlap in crude visceral extracts. Regarding the 35.0 kDa band, the molecular weight of the 35 kDa band is compatible with that reported for gastric aspartic proteases, such as fish pepsins, whose habitual electrophoretic displacement is recorded between 30.0 and 32.3 kDa [37].

In contrast to the bands, the signal observed at 48.3 kDa stood out as the most intense and dense in the profile. In a crude extract, this behavior does not indicate the hyperexpression of a large enzyme but rather reflects the intrinsic abundance of structural and conformational proteins from the visceral cellular matrix, such as actin or tropomyosin subunits. Nonetheless, a certain contribution from the heavy catalytic chains of marine collagenases, typically reported around 50 kDa, should not be ruled out [38]. Finally, the highest molecular weight band (69.1 kDa) may correspond to associated with remnant plasma proteins associated with the organs, whereas the lower band (15.8 kDa) evidences the presence of low-molecular-weight peptide fragments, derived either from the natural cleavage of collagenases (10 kDa subunit) or from partial autolysis processes occurring during the extraction stage.

Therefore, while molecular weight estimations provide valuable associations, confirming the specific presence and location of active proteases within this electrophoretic profile would require further activity-based assays, such as a zymogram. Although the molecular weights estimated by SDS-PAGE provide only an approximate assessment, the concordance between the observed bands and database records, together with the detected proteolytic activity, supports the presence of endogenous enzymes with potential biotechnological relevance.

#### 2.3.2. Determination of the Molecular Weight

**Figure 2:**
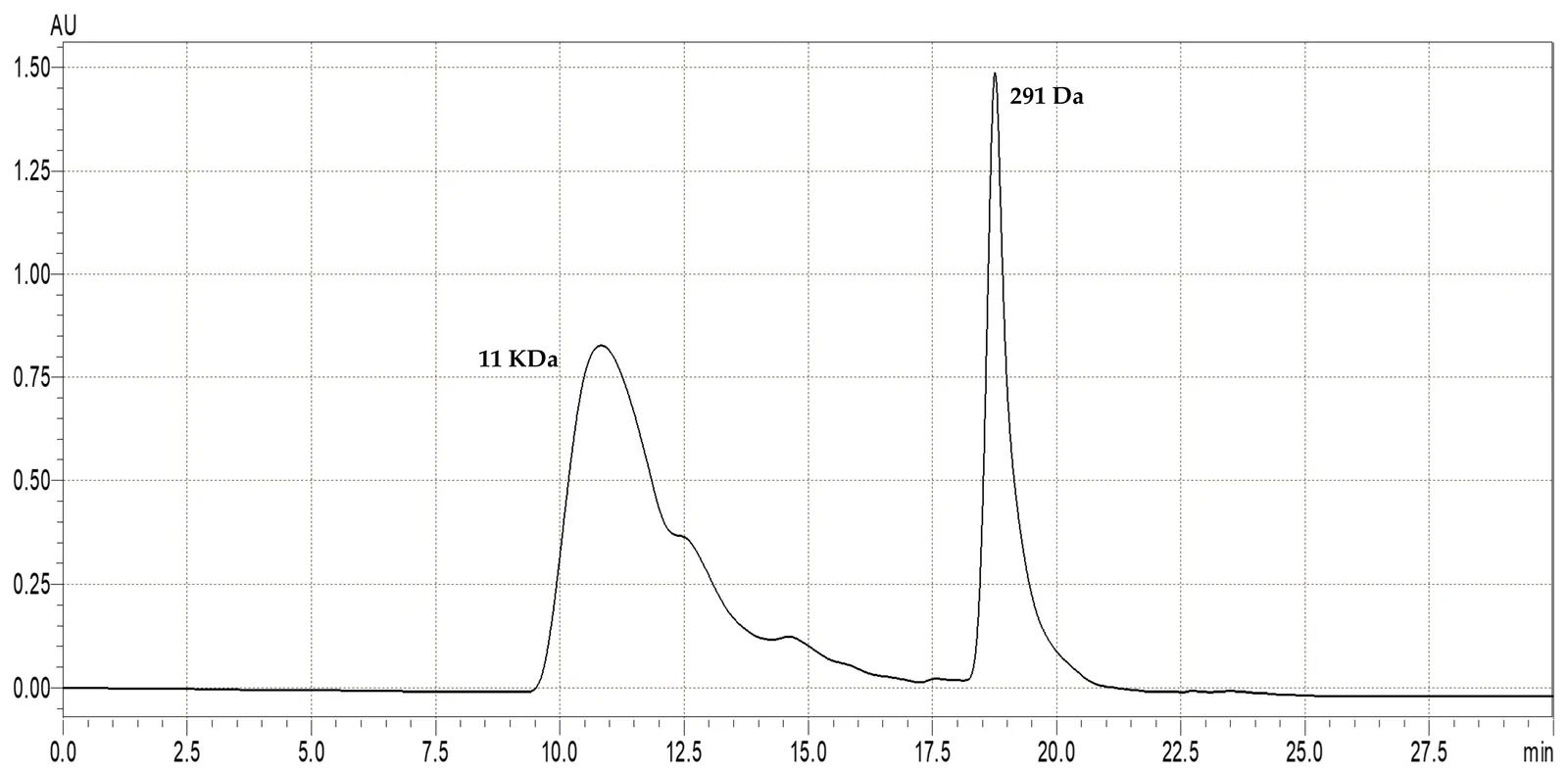
Molecular weight distribution profile of the protein hydrolysates determined by HPLC. Chromatogram of the hydrolysate produced with endogenous enzymes from viscera.

**Figure 3:**
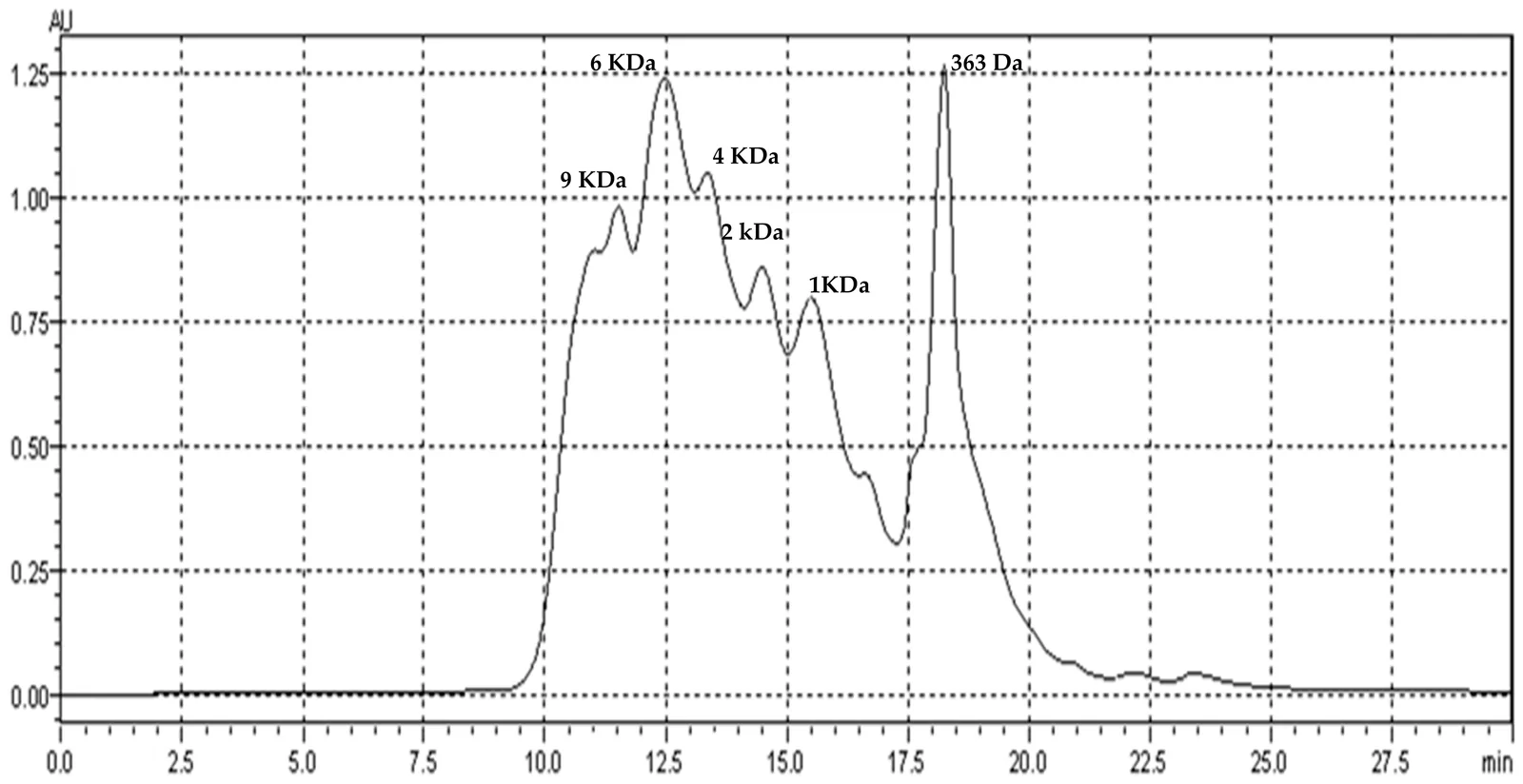
Molecular weight distribution profile of the protein hydrolysates determined by HPLC. Chromatogram of the hydrolysate produced with the commercial enzyme trypsin.

To understand the nature of the peptides generated during hydrolysis, the elution profiles of the different treatments were evaluated. Figure 2 and Figure 3 show the comparative chromatograms of the hydrolysates. This figure contrasts the differences in the degree of protein fragmentation, demonstrating how the use of the endogenous enzyme (Figure 2) produces a cleavage pattern distinct from that of a reference enzyme such as commercial trypsin (Figure 3).

As shown in Figure 2, the hydrolysate obtained using endogenous enzymes exhibited a bimodal elution profile with limited accumulation of intermediate fragments. The first broad peak, eluting at around 11 min, corresponds to partially hydrolysed proteins and large peptides with an estimated molecular weight of approximately 11 kDa.

Following clear baseline separation, a second, well-defined peak was observed at around 18.5 min. This represents a highly concentrated fraction of low-molecular-weight peptides (presumably dipeptides), around 291 Da. This polarised distribution is indicative of the combined action of endopeptidases and exopeptidases, which promotes the generation of small peptides and free amino acids while limiting the accumulation of stable intermediate species [39].

In contrast, the commercial trypsin hydrolysate (Figure 3) shows a heterogeneous elution profile. Instead of separate and defined fractions, a broad continuum of overlapping peaks spans from 10 to 20 min. The elution profile demonstrates the progressive degradation of the protein matrix into a wide range of intermediate-sized peptides, with the elution of distinct fractions corresponding to approximately 9 kDa, 6 kDa, 4 kDa, 2 kDa, and 1 kDa, finishing with a pronounced peak at 363 Da. These results reflect the specific ‘chromatographic fingerprint’ of trypsin; while some proteases generate only a few large fractions, trypsin typically produces numerous well-separated peaks covering a wide range of polarities and sizes due to its specific endopeptidase action [40,41].

### 2.4. Enzymatic Hydrolysis

The production of protein hydrolysates with bioactive potential and subsequent functional application largely depends on the protein content of the raw material, the type of proteolytic enzyme employed, and the extraction and purification conditions used for the enzymes.

To evaluate the efficacy of the endogenous extract as a sustainable biocatalyst, its performance was compared against commercial bovine trypsin, a well-characterized industrial enzyme known for its high specificity toward basic amino acid residues.

**Figure 4:**
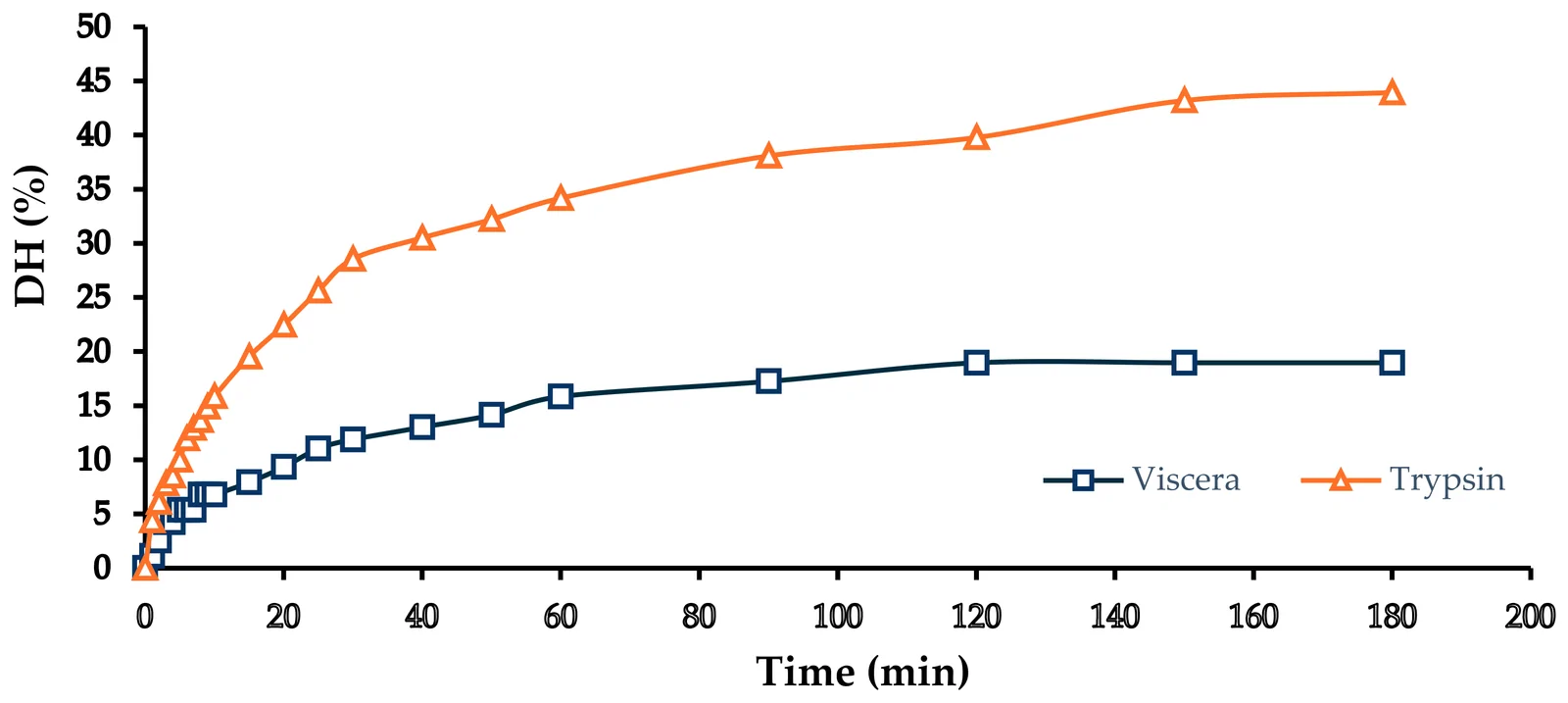
Enzymatic hydrolysis of fish bones using endogenous enzymes extracted from yellowfin tuna viscera and trypsin.

Under the optimal conditions previously established (pH 8, 40 °C, 180 min), the hydrolysis of yellowfin tuna tail bones showed notable differences between the two enzymatic systems (Figure 4). The process using the endogenous enzyme extracted from tuna viscera achieved a degree of hydrolysis (DH) of 18.96%, whereas the hydrolysis performed with commercial bovine trypsin resulted in a significantly higher DH (43.94%).

It is important to note that the experimental design did not aim to compare the enzymes on an equipotent basis. Instead, the dosages were selected to reflect their respective practical applications: 650 U/g of substrate for the endogenous extract (representing its optimized volumetric dosage) and 250 U/g for commercial trypsin (corresponding to a standard industrial dosage). Therefore, the differences observed in DH should be interpreted considering these distinct application strategies and enzyme characteristics, rather than being attributed exclusively to differences in catalytic specificity.

The higher DH observed with trypsin may be attributed to a combination of factors, including differences in enzymatic dosage, specific activity under the reaction conditions, and its well-defined catalytic specificity toward peptide bonds, particularly those involving the carboxyl groups of lysine and arginine residues, resulting in smaller peptides with more defined sequences [42]. Consequently, the higher hydrolytic efficiency observed for trypsin may partially reflect the higher catalytic efficiency of this purified enzyme system, although the influence of the different enzyme loads cannot be excluded.

However, despite being lower than the commercial enzyme, the DH achieved using the endogenous extract falls within a highly competitive range compared with other endogenous autolytic processes (Table 1).

As established in the literature, endogenous fish enzymes can yield a wide range of DH, from low values (5–15% in short periods) to highly extensive hydrolysis (>70%) during prolonged autolysis. However, the lack of parameter control or the use of substrates with low enzymatic load severely limits their immediate catalytic efficiency. For example, the natural autolysis of *Auxis rochei* viscera at 30 °C without pH control resulted in a DH of only 9.0% [43], and the autolysis of *Perna viridis* at 50 °C for 5 h reached 15.84% [44].

Conversely, the controlled modulation of pH, temperature, and time allows for an optimized DH range. In this context, the DH of 18.96% obtained in this study using the endogenous peptidase extract on yellowfin tuna bones in just 180 min at pH 8.0 and 40 °C is noteworthy. Achieving this level of hydrolysis on a recalcitrant substrate such as bone tissue in such a short time demonstrates the catalytic capacity of endogenous serine endopeptidases when operating under optimal alkaline conditions.

Although other endogenous processes can reach higher DH values, they demand significantly longer incubation periods or softer substrates. For instance, the autolysis of parrotfish heads at pH 9.0 achieved 30.65% but required 24 h of incubation [45]. Similarly, in *O. mykiss* viscera, optimized autolysis reached 68.8% in 7 h [46], and acid silage at pH 4 reached 75.8% but required 168 h [42]. Although commercial trypsin under the same conditions (pH 8.0, 40 °C, 180 min) yielded a higher DH (43.94%), the use of endogenous enzymes avoids the high costs associated with commercial proteases.

The DH, together with the sequence and size of the released peptides, can significantly influence the functional and bioactive properties of the hydrolysates. In this regard, the difference in the DH suggests distinct functional applications. While high DH values are typically associated with high solubility and smaller peptides, they can often result in bitter flavors due to the exposure of hydrophobic amino acids [47]. Conversely, moderate DH levels have been reported to preserve superior interfacial properties, including emulsifying and foaming capacities, compared with extensively hydrolyzed proteins [48,49]. Therefore, the moderate DH achieved with the endogenous extract may represent an advantageous characteristic for applications where preservation of peptide functionality and sensory attributes is required, although further studies are needed to confirm these properties.

Peptide yield was not determined in the present study; however, this parameter, together with peptide size distribution and sequence characterization, should be considered in future investigations to provide a more comprehensive evaluation of hydrolysis efficiency and functional potential.

Ultimately, the use of endogenous enzymes from yellowfin tuna may represent an alternative strategy to reduce dependence on commercial proteases while contributing to the valorization of tuna processing by-products. However, additional studies comparing equivalent enzymatic loads and evaluating peptide yield are required to fully assess the relative efficiency of endogenous and commercial enzyme systems.

### 2.5. Determination of Biological Activity and Toxicological Profile

Table 2 shows the biological activity results of the hydrolysates obtained using endogenous enzymes and commercial trypsin, demonstrating that both hydrolysates exhibited antioxidant activity, ACE-inhibitory capacity, and DPP-IV inhibitory activity.

#### 2.5.1. Antioxidant Activity

In the ABTS assay, the hydrolysate obtained using endogenous enzymes showed an antioxidant capacity of 10.1 ± 0.12 mg Trolox equivalents per gram (TE/g). In comparison, the hydrolysate produced with commercial trypsin exhibited slightly higher activity, reaching 11.8 ± 0.04 mg TE/g (Table 2). These results indicate that enzymatic hydrolysis enhances the antioxidant potential of tuna bone proteins, although the magnitude of this effect depends on the enzyme system employed and the characteristics of the generated peptides.

While most existing studies focus on muscle tissue or the use of expensive commercial catalysts, recent studies on fish processing by-products have demonstrated that skeletal fractions can also serve as sources of antioxidant peptides after enzymatic treatment. For instance, it has been reported that skipjack tuna (*Katsuwonus pelamis*) hydrolysates, obtained with tuna-derived trypsin, achieved a reducing power of 6.54 mg TE/mg protein, demonstrating the high efficiency of enzymes in releasing radical peptides [50]. Likewise, in a study on redlip mullet (*Chelon haematocheilus*) hydrolysates and other fishery by-products [51], it was noted that frames demonstrated that while non-hydrolyzed samples exhibit low ABTS activity (around 22.46%), enzymatic hydrolysis can increase this activity to over 93%. This trend agrees with the increase in antioxidant capacity observed for yellowfin tuna bone hydrolysates in the present study.

Furthermore, research on other underutilized species, such as the armoured catfish, has shown antioxidant activities of up to 174.7 TE/g in ABTS assays (approximately 43.72 mg TE/g) [52]. Although the activity in tuna bones is lower than that of muscle-rich catfish biomass, the results remain relevant considering the lower protein accessibility and higher mineral content typically associated with fish skeletal tissues.

Regarding the DPPH radical scavenging activity, the hydrolysate produced with commercial trypsin exhibited a significantly higher capacity (13.3 ± 0.40 mg TE/g) than that obtained with endogenous enzymes (7.3 ± 0.21 mg TE/g), as shown in Table 2. This increase in bioactivity after hydrolysis is consistent with recent reports on fish osseous fractions. For instance, studies on tuna frames have shown that while non-hydrolyzed samples exhibit negligible DPPH inhibition (3.73%), enzymatic treatment can boost this activity up to 18.27% [51]. This trend is further supported by Putra et al. (2024) [53], who reported a DPPH antioxidant capacity of 0.9141 ± 0.06 mmol TE/g (approx. 228.7 mg TE/g) in viscera (*Scomber scombrus*). The higher values reported for viscera-derived hydrolysates are likely related to their higher protein content and the presence of more accessible bioactive compounds compared with mineral-rich bone matrices.

Although the antioxidant capacity observed in tuna bone hydrolysates was lower than that reported for protein-rich viscera, the increase obtained after enzymatic treatment demonstrates that bone-derived proteins can be converted into antioxidant hydrolysates. The higher DPPH activity obtained with trypsin may be associated with its greater degree of hydrolysis (43.94%) and its ability to generate smaller peptide fractions; however, this relationship should not be interpreted as exclusively dependent on DH, since peptide composition and sequence also play a critical role in antioxidant mechanisms.

This behavior could be explained by the nature of the peptides generated under each treatment. It has been indicated that smaller peptides exhibit the highest antioxidant capacity, a result consistent with the effect observed in our trypsin-generated hydrolysates, where both peptide size and composition directly influence activity. Trypsin typically generates smaller peptides with a greater tendency to include hydrophobic residues, which would favor interaction with the DPPH radical, which is soluble in organic solvents [54].

In contrast, endogenous enzymes generate a more complex peptide mixture due to their broader proteolytic specificity, which may explain differences between antioxidant assays. The higher response of endogenous hydrolysates in some biological assays observed in this study further supports that bioactivity is influenced not only by hydrolysis extent but also by peptide specificity and diversity [55].

The results suggest that the antioxidant activity of the hydrolysates does not depend solely on the DH but is also influenced by peptide composition, particularly the presence of aromatic and hydrophobic residues, as well as molecular weight distribution [56].

Therefore, although trypsin hydrolysates showed higher ABTS and DPPH activities, the endogenous enzyme system remains a valuable alternative due to its ability to generate hydrolysates with distinct bioactive profiles. Further peptide characterization would be required to identify the specific sequences responsible for these antioxidant effects.

#### 2.5.2. Determination of ACE-Inhibitory Activity

Angiotensin-converting enzyme (ACE) is key in blood pressure regulation, as it produces angiotensin II (a vasoconstrictor) and inactivates bradykinin (a vasodilator) [57,58]. Consequently, its inhibition represents a fundamental strategy for managing hypertension, a context in which peptides derived from protein hydrolysates have recently gained interest [59]. Table 2 shows the angiotensin I-converting enzyme (ACE) inhibitory activity of the hydrolysates obtained using endogenous enzymes and commercial trypsin.

The hydrolysate obtained with endogenous visceral enzymes exhibited an IC_50_ value of 0.008 ± 0.004 mg/mL, whereas the hydrolysate produced with commercial trypsin exhibited an IC_50_ value of 0.010 ± 0.007 mg/mL. Although the endogenous hydrolysate presented a lower mean IC_50_ value, no statistically significant differences (*p* > 0.05) were observed between the two hydrolysates. Nevertheless, both hydrolysates exhibited remarkably low IC_50_ values, indicating a strong ACE-inhibitory activity. In other tuna and sardine by-products (including head, muscle, and viscera), IC_50_ values between 0.24 and 1.16 mg/mL [60] have been found; similarly, ribbon fish visceral residues treated with Alcalase, Flavourzyme, and Papain reported values of 0.902, 1.32, and 1.56 mg/mL, respectively [61]. The exceptionally low IC_50_ values obtained in the present study indicate highly potent ACE-inhibitory activity, surpassing many of the values reported in the literature for hydrolysates generated from fish processing residues.

These differences may be attributed to variations in raw material composition, enzyme specificity, hydrolysis conditions, and the molecular weight distribution of the generated peptides. Furthermore, previous studies have shown that peptide purification, particularly by membrane fractionation, can substantially enhance ACE-inhibitory activity, often yielding IC50 values below 0.5 mg/mL [60,62]. Therefore, the already highly potent activity observed in the crude hydrolysates suggests that additional purification strategies could further isolate these highly active sequences.

Although the endogenous enzyme hydrolysate exhibited a lower mean IC_50_ value than the commercial trypsin hydrolysate, no statistically significant differences were detected between the two treatments. Interestingly, the endogenous hydrolysate achieved comparable ACE-inhibitory activity despite its lower DH (18.96%) compared with the trypsin hydrolysate (43.94%). This indicates that ACE inhibition is not exclusively dependent on the extent of hydrolysis but is strongly influenced by enzyme specificity and the structural characteristics of the released peptides. ACE-inhibitory peptides are commonly associated with low-molecular-weight fractions and the presence of specific amino acid residues, particularly hydrophobic and aromatic residues that favor interactions with the ACE active site. In this context, the 291 Da fraction detected in the endogenous hydrolysate HPLC profile (Figure 2) represents a molecular weight range compatible with the presence of small bioactive peptides. However, further peptide sequencing approaches would be required to confirm the identity and contribution of specific ACE-inhibitory sequences.

The endogenous visceral extract is expected to contain multiple proteolytic activities, which may contribute to the generation of a broader diversity of peptide fragments than commercial trypsin alone [63,64]. This broader proteolytic specificity may favor the production of peptides with structural features associated with ACE inhibition compared with commercial trypsin, whose strictly endopeptidase specificity for Lys/Arg residues produces less diverse and potentially larger fragments [65]. In addition, the presence of collagen in the bones, which is rich in glycine and proline [66], may further contribute to the formation of peptide fragments with structural characteristics suitable for ACE inhibition. These factors may explain the tendency toward lower IC_50_ values observed for the endogenous hydrolysate, although the differences between treatments were not statistically significant.

Taken together, these results indicate that endogenous visceral enzymes can produce hydrolysates with ACE-inhibitory activity comparable to that obtained with commercial trypsin, despite achieving a lower DH. This finding highlights that peptide bioactivity is governed not only by hydrolysis extent but also by the specificity of the enzymatic system and the characteristics of the generated peptides. These results support the potential application of endogenous tuna visceral proteases as sustainable biocatalysts for producing antihypertensive peptides from fish-processing by-products.

#### 2.5.3. Determination of DPP-IV Inhibitory Activity

The inhibition of DPP-IV is physiologically relevant because it helps preserve GLP-1 levels, thereby stimulating insulin secretion and contributing to improved glycemic control [67]. DPP-IV inhibitors such as sitagliptin, saxagliptin, linagliptin, alogliptin, and vildagliptin have demonstrated strong clinical efficacy, reducing glycated hemoglobin (HbA1c) levels by approximately 0.5–0.8% in patients with type 2 diabetes, while exhibiting excellent tolerability and cardiovascular safety [68]. Currently, DPP-IV inhibitors are well established as part of individualized management for patients with type 2 diabetes, particularly recommended for those with a low risk of hypoglycemia or as a second-line therapy following metformin use [69].

Table 2 shows a more pronounced DPP-IV inhibitory activity in the hydrolysate treated with trypsin (0.83 ± 0.37) than that obtained using endogenous enzymes (1.60 ± 0.47). In the context of crude fish hydrolysates, these values are highly competitive.

Generally, crude fish by-product hydrolysates show IC_50_ values in the range of 1 to 5 mg/mL, typically requiring concentrations between 5 and 10 mg/mL to achieve inhibition levels of 40% to 70% [70,71]. In contrast, fractions purified through methods such as ultrafiltration or chromatography reach significantly higher potencies in the micromolar (5–300 µM) or nanomolar range [72]. These reference values are consistent with those reported for complex mixtures such as blue whiting (2.12–2.90 mg/mL) [73], yellowtail gelatin (1.19 mg/mL) [70], and silver carp (1.12 mg/mL) [74].

Furthermore, it is noteworthy that the trypsin-generated hydrolysate (0.83 mg/mL) falls within the range of high-potency substrates such as hydrolyzed camel milk (0.52–1.52 mg/mL) [75] and bovine dairy isolates (0.66–1.59 mg/mL) [76]. This potency is even superior to several plant-based proteins (hemp, pea, rice, and soy), which typically exhibit IC_50_ values between 0.73 and 3.5 mg/mL [77,78].

The superior inhibitory capacity of commercial trypsin is consistent with its degree of hydrolysis and the resulting molecular weight distribution observed in Figure 3. While the endogenous extract’s bimodal profile retained a substantial protein fraction centered at 11 kDa, trypsin’s action produced a broad continuum of intermediate-sized peptides (1–9 kDa). This heterogeneous distribution likely ensures a higher density of bioactive sequences capable of interacting with the DPP-IV active site. Specifically, the higher fragmentation degree may favor the exposure of key residues such as proline, alanine, or glycine at the penultimate N-terminal position, a critical structural requirement for high-affinity binding and effective inhibition of this enzyme [70,79].

#### 2.5.4. Toxicological Profile Evaluation

During the hatching of *Artemia salina*, young, motile, and healthy nauplii were obtained after 36 h of incubation, corresponding to the organism’s most sensitive larval phase to bioactive compounds. This acute toxicity evaluation was performed in triplicate and repeated in two independent assays to ensure reproducibility. In all trials, the distribution of organisms was highly homogeneous, with an average of 10 ± 1 nauplii per well.

Assay validity was confirmed by the control groups. The negative control (4% artificial seawater) exhibited a minimal mean mortality of 1.66% ± 4.15%, well within the acceptable biological range, whereas the positive control (K_2_Cr_2_O_7_ at 400 ppm) caused 100% mortality.

Regarding the yellowfin tuna bone hydrolysates, concentrations ranging from 50 to 2400 μg/mL were evaluated for both endogenous enzyme and commercial trypsin treatments. Across the two independent assays, the hydrolysates demonstrated a remarkably high safety profile. In one of the assays, 0.00% ± 0.00% mortality was recorded across all concentrations. In the other assay, a minimal mortality of 3.33% ± 5.77% was observed only at 100 and 400 μg/mL for both samples. This standard deviation reflects the death of a single nauplius in one of the three replicate wells (well mortalities of 0%, 0%, and 10%). The high level of agreement between the two independent assays indicates good reproducibility, with the slight discrepancy being attributable to the inherent biological variability of the *Artemia salina* model rather than to a concentration-dependent toxic effect. The absence of a consistent mortality pattern across concentrations further supports the non-toxic nature of the hydrolysates under the conditions tested.

Because this negligible mortality lacked a dose-dependent trend and remained well below the 10% toxicity threshold, it is not considered biologically significant and is likely attributed to natural nauplii sensitivity or handling. Additionally, while the solubility of the extracts decreased above 1200 μg/mL, no toxic effects were identified even at the highest concentrations. Taken together, these results suggest that the peptide hydrolysates obtained do not exhibit acute toxicity against *Artemia salina* within the studied concentration range, representing a favorable property in terms of biological safety and potential food, nutraceutical, or pharmaceutical applications.

The results of this study demonstrate the feasibility of using endogenous visceral enzymes as a sustainable biocatalyst for generating bioactive hydrolysates from yellowfin tuna by-products. Future studies should focus on the further characterisation and standardisation of the enzymatic extract, taking into account the potential variability associated with biological sources and independent batches. Additionally, larger-scale investigations, including process optimisation, enzyme stability and economic evaluation, will be valuable in assessing the feasibility of industrial implementation. Further complementary in vitro and in vivo safety evaluations, together with bioavailability studies, will contribute to a more comprehensive understanding of the potential applications of these hydrolysates.

## 3. Materials and Methods

### 3.1. Chemicals and Reagents

All experimental procedures were conducted utilizing analytical-grade reagents. potassium phosphate monobasic and dibasic, sodium chloride, Tris-HCl (tris(hydroxymethyl)aminomethane hydrochloride), and trichloroacetic acid, obtained from Thermo Fisher Scientific (Waltham, MA, USA). Dextrose, casein, SDS (sodium dodecyl sulfate), TEMED (*N*,*N*,*N*′,*N*′-tetramethylethylenediamine), Tricine, Sephadex G-100, and Sephadex G-25 resin were obtained from Sigma Aldrich (St. Louis, MO, USA), while ammonium sulfate, 2-mercaptoethanol, glycerol, sodium hydroxide were purchased from Merck KGaA (Darmstadt, Germany). Ammonium persulfate, Bromophenol brilliant blue, Coomassie Brilliant Blue, acrylamide, and bisacrylamide were obtained from Bio-Rad (Hercules, CA, USA). Bovine hemoglobin, trichloroacetic acid, glycine, ammonium sulfate, bovine serum albumin, aprotinin, vitamin B12, norleucine, and dipeptidyl peptidase IV (DPP-IV) from porcine kidney were procured from Sigma Chemical Co. (St. Louis, MO, USA). The commercial protease, Trypsin were generously supplied by Sigma-Aldrich (St. Louis, MO, USA). Angiotensin-converting enzyme (ACE, EC 3.4.15.1) was also sourced from Sigma-Aldrich. Chromogenic substrates for DPP-IV (H-Gly-Pro-AMC·HBr and Z-Gly-Pro-AMC, respectively) were purchased from Bachem (Bubendorf, Switzerland).

### 3.2. Raw Materials

**Figure 5:**
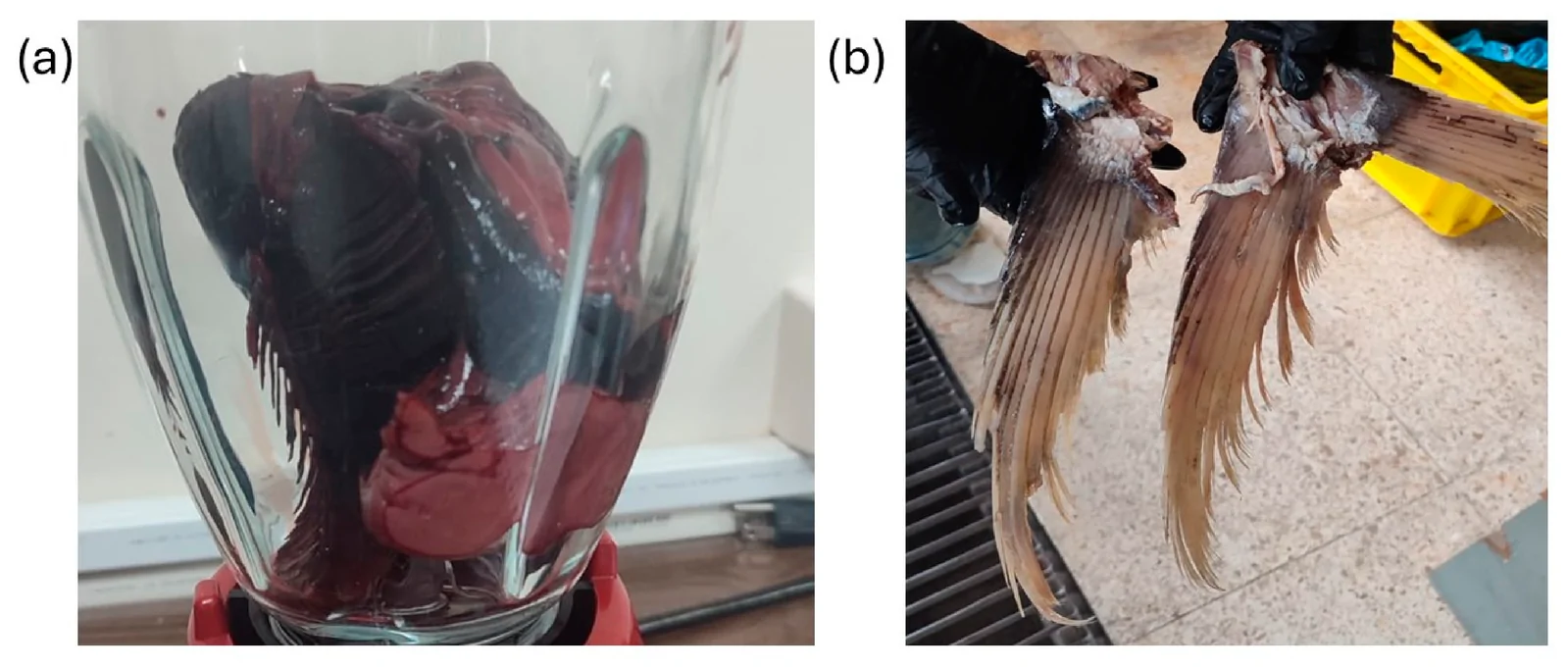
Yellowfin tuna (*Thunnus albacares*) by-products used in this study: (**a**) viscera collected for endogenous protease extraction and (**b**) tail frames used as the substrate for protein hydrolysis.

Viscera and frames (bones with attached muscle residues) of yellowfin tuna (*Thunnus albacares*) were obtained from a wholesale fish market in Quito, Ecuador. All raw materials were collected from the same supplier and corresponded to a single batch of freshly processed fish, purchased on the same day, to minimise biological variability. The by-products were transported to the laboratory in an ice-filled cooler immediately after collection to preserve their integrity and endogenous enzymatic activity. Upon arrival, the viscera were separated into anatomical fractions and immediately processed for enzyme extraction, while the frames were chopped and stored at −80 °C until required. All hydrolysis experiments were performed using aliquots derived from this same batch of raw material to ensure experimental reproducibility. Representative images of the yellowfin tuna by-products used in this study are shown in Figure 5, including the viscera used for endogenous protease extraction (Figure 5a) and the tail frames used as the substrate for hydrolysate production (Figure 5b).

### 3.3. Extraction and Characterization of Proteolytic Enzymes from Yellowfin Tuna Viscera

#### 3.3.1. Substrate Preparation

For the enzymatic extraction, 100 g of the fresh viscera were immediately subjected to cold mechanical homogenization in the presence of Tris–HCl buffer (10 mM, pH 8) supplemented with CaCl_2_, at a 1:5 (*w*/*v*) ratio (100 g of viscera in 500 mL of cold buffer). This was performed using a commercial blade blender (Oster, 700 W, 120 V, 1.25 L capacity, Quito, Ecuador) for 5 min. The pH was adjusted as necessary using HCl (0.1 M). The mixture was centrifuged at 5000× *g* for 30 min at 4 °C (MPW Med. Instruments, Warsaw, Poland), and the supernatant containing the enzymatic extract was collected and stored at −80 °C until purification by salt precipitation.

Subsequently, enzyme precipitation was carried out following the protocol described by Kholif et al. (2022) [80]. To this end, the enzymatic extract was mixed with ammonium sulfate until reaching a saturation of 60% (*w*/*v*), and the solution was stirred in a thermal bath to promote protein precipitation. The mixture was then refrigerated for 1 h and centrifuged at 10,000× *g* for 10 min. After centrifugation, the supernatant was discarded, and the precipitate was resuspended in Tris-HCl buffer (0.1 M, pH 8). The resuspended enzyme preparation was subsequently purified by Sephadex chromatography (G-25 and G-100), and its proteolytic activity was re-determined prior to the hydrolysis experiments. The activity of this purified enzyme preparation was used to calculate the enzyme dosage applied during hydrolysis.

#### 3.3.2. Determination of Proteolytic Activity

Proteolytic activity of the visceral extracts was determined using casein as the substrate, following the method described by Anson (1938) [81] with minor modifications.

The substrate was prepared by dissolving casein in phosphate buffer (50 mM, pH 7). The reaction mixtures contained 100 µL of enzymatic extract and 1.1 mL of 1% (*w*/*v*) casein solution. Blanks consisted of 1.8 mL of 10% TCA, 1.1 mL of 1% casein solution, and 100 µL of distilled water.

To evaluate the enzymatic behavior under representative conditions, reactions were incubated at temperatures ranging from 40 °C to 80 °C for 20 min, selected based on literature reports for marine digestive enzymes [82,83]. The reactions were stopped by adding 1.8 mL of 10% TCA. Subsequently, the mixtures were centrifuged at 3500× *g* for 20 min, and absorbance was measured at 280 nm. The spectrophotometer was zeroed using a 10% (*w*/*v*) TCA blank. One unit of proteolytic activity was defined as the amount of enzyme capable of producing an increase of 0.1 absorbance units under the experimental conditions described [84].

Based on these evaluations and a robust literature background, the operational conditions for the visceral enzymatic extract were established at pH 8.0 and 40 °C to evaluate the overall proteolytic activity of the multienzyme mixture. At this alkaline pH, acidic proteases potentially present in the crude extract (such as pepsins) are irreversibly inactivated, ensuring that the measured activity is predominantly driven by the collective action of alkaline proteases [85].

Considering its visceral origin, the enzymatic extract is expected to contain a complex mixture of digestive proteases whose overall activity is primarily driven by alkaline serine proteases. This assumption is supported by previous studies reporting that serine proteases constitute the dominant proteolytic fraction in crude fish visceral extracts and are responsible for their high catalytic performance under mildly alkaline pH and moderate temperature conditions, as reported by Ríos et al. and Montoya [18,86].

Consistently, Borges et al. (2023) [11], reported optimal proteolytic activity at pH 8.0–9.0 in multienzymatic visceral extracts from sardine (*Sardina pilchardus*), zebra blenny (*Salaria basilisca*), and bogue (*Boops boops*). Likewise, crude digestive protease preparations from Baltic herring exhibited sustained activity across a pH range of 7.5–8.8 [87]. These observations closely align with the activity profile obtained in the present study, reinforcing the contribution of alkaline serine proteases to the overall hydrolytic capacity of the extract.

Regarding temperature, the selection of 40 °C aligns with the general thermal behavior of marine digestive protease mixtures. Borges et al. (2023) [11] revealed that these crude extracts exhibit their highest collective stability at temperatures between 40 °C and 50 °C, experiencing a rapid loss of total proteolytic activity at 60 °C. This pool of marine enzymes typically exhibits high structural flexibility and elevated catalytic efficiency at moderate temperatures, but at the expense of lower overall thermal stability, explaining the drastic decrease in the extract’s activity exceeding 50 °C [88,89,90].

Additionally, the enzyme dosage required for the subsequent hydrolysis experiments was established based on the proteolytic activity of each enzymatic preparation, determined using a standard tyrosine calibration curve (1–100 µM). Commercial trypsin (Sigma-Aldrich, St. Louis, MO, USA) was evaluated alongside the endogenous visceral extract as a reference enzyme. The amount of tyrosine released at different enzyme concentrations was used to calculate the proteolytic activity, allowing the enzyme dosage to be expressed as units per gram of substrate (U/g). Since the endogenous preparation consisted of a crude enzymatic extract, whereas commercial trypsin was a purified enzyme, differences in enzyme purity and specific activity were expected. Consequently, the amount of each enzymatic preparation added during hydrolysis was adjusted according to its measured proteolytic activity to ensure an activity-based comparison between treatments.

### 3.4. Production of Peptide Hydrolysates from Yellowfin Tuna Bones

#### 3.4.1. Pretreatment and Sample Preparation

The chopped tail bones were initially washed in a 5% (*w*/*v*) NaCl brine solution to remove residual blood and impurities. A chemical cleaning step was subsequently performed to remove non-collagenous proteins and other soluble components by immersion in 0.1 N NaOH solution at a solid-to-liquid ratio of 1:5 (*w*/*v*) for 1 h under continuous agitation. This alkaline treatment was repeated twice.

Prior to hydrolysis, the bone samples were demineralized following the protocol of Ordóñez and Mosquera [91]. Samples were immersed in 0.5 M EDTA solution (pH 5.5), adjusted with 0.1 N HCl, and maintained under continuous agitation for 18 h. This procedure was repeated four times to ensure complete demineralization. The samples were then rinsed with distilled water for 30 min and subsequently treated with 0.05 M acetic acid overnight. After acid treatment, the samples were rinsed thoroughly with distilled water to remove residual acetic acid and then stored at −80 °C prior to freeze-drying, which was performed over a period of 3 days.

Once freeze-dried, the bones were ground using a hammer mill (Condux LHM 20/16, NETZSCH-CONDUX Mahltechnik GmbH, Hanau, Germany) followed by a knife mill (GRINDOMIX GM 200, Retsch GmbH, Haan, Germany) equipped with a 0.5 mm sieve to obtain a fine bone powder. The resulting flour was subjected to nutritional characterization following official AOAC methods [92]. Moisture content was determined according to AOAC method 934.01, ash content according to AOAC method 942.05, crude protein content by the Kjeldahl method following AOAC method 984.13, using a nitrogen-to-protein conversion factor of 5.55, and crude fat content was determined by Soxhlet extraction following AOAC method 920.39. The results were expressed in grams per 100 g of dry sample. Finally, the flour was stored under refrigeration until its use in the hydrolysis process.

#### 3.4.2. Enzymatic Hydrolysis

The pulverized flour obtained from the tail bones was hydrolyzed using either the endogenous enzymatic extract or commercial trypsin (Sigma-Aldrich, St. Louis, MO, USA). The substrate was prepared by dispersing 1 g of tail bone flour in 50 mL of distilled water, establishing a 1:50 (*w*/*v*) sample-to-liquid ratio. This suspension was homogenized and conditioned in a water bath until reaching the required temperature and pH for each treatment.

For hydrolysis with the endogenous enzyme, the extract was added at a volume of 10 µL per gram of substrate, which corresponds to an enzymatic activity of 650 U/g of substrate. The reaction was conducted under the conditions of pH 8 and 40 °C for 180 min.

For the comparative control using commercial trypsin, the hydrolysis was performed under optimal conditions of pH 8 and 40 °C for 180 min, maintaining the same 1:50 (*w*/*v*) sample-to-liquid ratio. The commercial enzyme was added at a dose of 20 mg per gram of substrate, corresponding to an activity of 250 U/g of substrate.

The pH was maintained constant using 0.1 M NaOH with a manual pH-Stat setup, consisting of a digital burette (Titrette, BRAND, Wertheim, Germany) and a pH meter (Orion Star A211, Thermo Scientific, Waltham, MA, USA) during the 180 min hydrolysis reaction, the pH was continuously monitored and manually adjusted by adding 0.1 M NaOH whenever a decrease in pH was detected due to peptide bond cleavage. A total volume of 2.10 mL of 0.1 M NaOH was consumed to maintain the reaction pH at 8.0 throughout the hydrolysis process. Post-hydrolysis, the enzymes were inactivated by heating the samples at 90 °C for 15 min. The mixtures were then centrifuged at 4200× *g* for 20 min at 4 °C (Beckman Coulter J2-MC, Indianapolis, IN, USA). The supernatants were collected, freeze-dried, and stored at −20 °C until further use. The degree of hydrolysis (DH) was calculated according to Adler-Nissen (1986) [93].

(1) $$\mathrm{DH}\left(\%\right)=\frac{B\;\times \;M\;\times 100}{\alpha \;\times {\;h}_{\mathrm{tot}}\times \;\mathrm{mp}\;\times \;p}$$

where:

B corresponds to the volume of 0.1 M NaOH consumed during the reaction (mL).

M is the normality of the NaOH solution, and (mp) is the mass of the sample (g).

α is the average degree of dissociation of the α-NH_2_ groups.

h_tot_ represents the total number of peptide bonds per protein equivalent (9.06 mEq/g).

p is the percentage of protein in the sample (%).

### 3.5. Molecular Characterization

#### 3.5.1. Determination of the Molecular Weight Profile of the Enzymatic Visceral Extract (SDS-PAGE)

Sodium dodecyl sulfate–polyacrylamide gel electrophoresis (SDS-PAGE) was performed to evaluate the protein profile of the crude enzymatic extract obtained from yellowfin tuna viscera [94]. A Mini-PROTEAN II system (Bio-Rad^®^, Hercules, CA, USA) was used. Electrophoresis was performed using a 10% polyacrylamide gel, suitable for resolving proteins with molecular weights up to approximately 100 kDa. The electrophoretic run was carried out at a constant current, starting at 30–40 mA while the samples migrated through the stacking gel, and subsequently increased to 80 mA for the separation phase until the tracking dye reached the bottom of the gel.

The crude extract samples were prepared in a loading buffer containing SDS, Tris, glycerol, bromophenol blue, and β-mercaptoethanol as a reducing agent, and subsequently heated at 90 °C for 10 min to ensure complete protein denaturation. A volume of 10 µL of each sample was loaded per well. A molecular weight marker ranging from 6.5 to 200 kDa (Sigma Marker S8445-10VL, Sigma-Aldrich, St. Louis, MO, USA) was used for molecular weight estimation. After electrophoretic separation, the gel was stained with Coomassie Brilliant Blue R-250 and subsequently destained using 10% acetic acid until a clear background was obtained, allowing visualization of the protein bands present in the crude extract.

The molecular weights of the protein bands were determined by calculating the relative mobility (Rf) of each band and fitting it to a logarithmic standard curve (log_10_ *MW* vs. Rf) generated from the commercial marker (R^2^ = 0.9989).

The molecular weights of the obtained bands were correlated with information available in the BRENDA database [95]. This correlation enabled the inference of the possible identity of the enzymes present, based on the correspondence between the experimental molecular weights and those reported in the literature for proteases with similar characteristics.

#### 3.5.2. Determination of the Molecular Weight

The molecular weight distribution of the peptide hydrolysates was analyzed using size-exclusion chromatography following the method described by Martínez-Álvarez et al. (2012) [96]. Separation was achieved on a Peptide PC 3.2/30 column (GE Healthcare, Barcelona, Spain), coupled to an HPLC Nexera (Shimadzu, Tokyo, Japan). Prior to injection, hydrolysate samples (10 mg/mL) were filtered through 0.45 μm syringe filters. The mobile phase consisted of 30% (*v*/*v*) acetonitrile in Milli-Q water supplemented with 0.1% (*v*/*v*) trifluoroacetic acid (TFA), maintained at a constant flow rate of 0.1 mL/min. Peptide elution was monitored spectrophotometrically at 214 nm and 280 nm. A calibration curve was constructed using molecular weight standards, including aprotinin (6511 Da), vitamin B12 (1345 Da), hippuryl-histidyl-leucine (429 Da), and glycine (75 Da), while bovine serum albumin (BSA, 67,000 Da) was utilized to establish the column’s void volume. The relative proportion of peptides within specific molecular weight ranges was determined by integrating the respective peak areas.

### 3.6. Determination of Biological Activity

The bioactivity of the hydrolysates was evaluated by determining their inhibitory activities against dipeptidyl peptidase IV (DPP-IV) and angiotensin I-converting enzyme (ACE) [97], followed by the assessment of antioxidant activity using the ABTS [98] and DPPH [99,100] assays.

The enzymatic inhibition assays (ACE and DPP-IV) were performed at a final concentration of 1 mg/mL in the reaction mixture. In contrast, the antioxidant capacities were evaluated using appropriate sample dilutions, and the results were expressed per milligram of hydrolysate. All determinations were performed in triplicate *(n* = 3).

#### 3.6.1. Determination of DPP-IV Inhibition Assay

Dipeptidyl peptidase IV (DPP-IV) inhibitory activity was evaluated using a fluorometric assay performed in black 96-well microplates, following the procedure described by Bougatef et al. (2023) [101]. The samples were previously freeze-dried and reconstituted in 100 mM Tris–HCl buffer (pH 8). For the assay, 30 µL of sample solution at 10 mg/mL or at different concentrations was mixed with 20 µL of DPP-IV enzyme solution (10.64 mU) and 150 µL of Tris–HCl buffer, resulting in a final reaction volume of 200 µL and a sample-to-enzyme ratio of 1:50. The reaction mixtures were incubated at 37 °C for 15 min. Subsequently, the assay was initiated by adding 100 µL of reaction buffer containing the fluorogenic substrate H-Gly–Pro–AMC·HBr at a final concentration of 25 µM. Fluorescence was recorded every minute for 15 min using a microplate reader, with excitation and emission wavelengths set at 340 and 440 nm, respectively.

DPP-IV inhibitory activity was determined by comparing the initial fluorescence slopes obtained in the absence (control) and presence of the samples, and the results were expressed as IC_50_ values (amount of sample necessary to produce a 50% enzymatic inhibition). There were three replicates per sample.

#### 3.6.2. Determination of ACE Inhibition Assay

The ability of peptide fractions to inhibit angiotensin I-converting enzyme (ACE) was evaluated following the method described by Bougatef et al. (2023) [101], with modifications.

The assay was performed in a black 96-well microplate. For the initial inhibition assessment, 30 µL of the sample, 150 µL of working buffer (150 mM Tris-HCl, pH 8.3, containing 1.125 M NaCl), and 20 µL of ACE were added to each well. Controls were prepared by using 180 µL of working buffer and 20 µL of ACE (without sample), and blanks containing buffer and ACE inactivated with 5 M HCl were also included.

The plate was pre-incubated for 15 min at 37 °C, and the reaction was initiated by adding 100 µL of the fluorogenic substrate Abz-Gly-Phe (NO_2_)-Pro (0.45 mM) in working buffer. The final sample concentration was adjusted to 1 mg/mL, for a total reaction volume of 300 µL per well. Fluorescence was recorded at 360/400 nm (excitation/emission) every 1 min for 15 min using a Clariostar microplate reader (BMG Labtech, Ortenberg, Germany). The ACE-inhibitory activity of the hydrolysates was tested at different concentrations and expressed as IC_50_, defined as the concentration of hydrolysate required to inhibit 50% of ACE activity. There were three replicates per sample.

#### 3.6.3. Determination of Antioxidant Activity Using the ABTS Method

Antioxidant activity was determined using the ABTS radical cation decolorization assay, as described by Re et al. (1999) [93,98]. The ABTS stock solution was prepared by dissolving ABTS in distilled water at a concentration of 7 mM and adding potassium persulfate (K_2_S_2_O_8_) at a concentration of 2.45 mM. The mixture was kept in the dark at room temperature for 16 h to allow radical formation.

Prior to analysis, the ABTS stock solution was diluted with distilled water to obtain an absorbance of approximately 0.70 at 734 nm, ensuring adequate sensitivity for antioxidant detection. For the assay, 10 µL of sample was mixed with 290 µL of the ABTS working solution and incubated at 30 °C for 10 min in the dark. Absorbance was then measured at 734 nm.

Antioxidant activity was quantified using calibration curves prepared with Trolox and ascorbic acid as reference standards. Results were expressed as milligrams of Trolox equivalents per gram of hydrolysate (mg TE/g) and milligrams of ascorbic acid equivalents per gram of hydrolysate (mg AAE/g).

#### 3.6.4. Determination of Antioxidant Activity Using the DPPH Method

Antioxidant activity was determined using the DPPH radical scavenging assay, as described by Brand-Williams et al. (1995) [99]. For the DPPH method, a DPPH stock solution (0.0591 mg/mL) was prepared by dissolving 5.91 mg in 100 mL of methanol: water (80:20 *v*/*v*) and sonicating for 20 min.

Trolox and ascorbic acid were used as standards. The Trolox stock was prepared by dissolving 10.2 mg in 50 mL of absolute ethanol, resulting in a final concentration of 0.204 mg/mL. The ascorbic acid stock solution was prepared by dissolving 2.5 mg in 25 mL of distilled water to obtain a concentration of 0.1 mg/mL.

In a 96-well microplate, 20 µL of each sample or standard and 180 µL of the DPPH solution were added. The blank consisted of 20 µL of distilled water and 180 µL of methanol: water (80:20 *v*/*v*), while the control was prepared with 20 µL of distilled water and 180 µL of the DPPH solution. The absorbance was measured at 515 nm for 40 min at 25 °C.

The radical inhibition was calculated using the following equation:

(2) $$\mathrm{DPPH}\;\mathrm{quenched}\;\left(\%\right)=1-\left(\frac{A_{\mathrm{sample}}-A_{\mathrm{blanck}}}{A_{\mathrm{control}}-A_{\mathrm{blank}}}\right)\times 100$$

The antioxidant capacity of the samples was expressed as milligrams of Trolox or ascorbic acid equivalents per gram of hydrolyzed sample, according to the interpolation of the respective calibration curves.

### 3.7. Toxicological Profile Evaluation

The *Artemia salina* toxicity assay was performed in sterile flat-bottom 96-well plates (Costar 3590), following a protocol adapted from Meyer et al. (1982) [102].

Cysts of *Artemia salina* from Mackay Marine were incubated under continuous white light at 28 °C in 1 L of distilled water containing 40 g/L sea salt and 6 mg of yeast. The medium pH was maintained between 7.5 and 8.5 with constant aeration. After 36 h of hatching, nauplii were collected by positive phototaxis and distributed into 96-well plates.

In each experimental well, 10 ± 2 nauplii were inoculated in 100 µL of 4% artificial seawater, along with 100 µL of sample at different concentrations (prepared in artificial seawater from hydrolysates at 10 mg/mL). The positive control consisted of 100 µL of potassium dichromate (K_2_Cr_2_O_7_) at 400 ppm, and the negative control consisted of 100 µL of artificial seawater. Peripheral wells were filled with 200 µL of artificial seawater to minimize evaporation. The plates were incubated at 28 °C under white light for 24 h.

At the end of the exposure period, observations were performed under a stereomicroscope (Olympus SZX16; Olympus Corporation, Tokyo, Japan), considering nauplii as dead when immobile for at least 10 s. The mortality percentage was calculated using the following formula:

(3) $$\mathrm{Mortality}\;\left(\%\right)=\frac{N_{\mathrm{dead}}}{N_{\mathrm{total}\;\mathrm{nauplii}}}\times 100$$

### 3.8. Statistical Analysis

All experimental determinations were performed in triplicate (*n* = 3), and the data were expressed as the mean ± standard deviation (SD). To evaluate the statistical differences between the two treatments (endogenous tuna protease viscera extract versus commercial trypsin), a pairwise comparison was conducted using an independent samples Student’s *t*-test. Differences between means were considered statistically significant at *p* < 0.05.

## 4. Conclusions

The bones of yellowfin tuna, characterized by high protein and low lipid content, constitute a suitable raw material for the production of protein hydrolysates. Hydrolysis using endogenous proteases from tuna viscera generated hydrolysates with a distinct peptide profile, highlighting a low-molecular-weight fraction (291 Da). While commercial trypsin achieved a higher degree of hydrolysis, the endogenous extract yielded hydrolysates with measurable antioxidant and DPP-IV inhibitory activities, alongside an exceptionally potent ACE-inhibitory capacity. The hydrolysates showed no evidence of acute toxicity in the *Artemia salina* model under the experimental conditions evaluated, supporting their favorable performance in this preliminary toxicity screening assay. These findings support the feasibility of using endogenous visceral proteases for the valorization of tuna processing by-products within a circular bioeconomy framework, positioning these peptide-rich ingredients as highly promising candidates for cardiovascular health applications. However, the *Artemia salina* assay represents only an initial assessment of acute toxicity and is not sufficient to establish safety for food or nutraceutical applications. Therefore, further studies, including simulated gastrointestinal digestion, identification of bioactive peptides, cytotoxicity assays using mammalian cell lines, genotoxicity assessment (e.g., Ames test), in vivo toxicity studies, bioavailability, and stability evaluations, are required to comprehensively assess their safety and confirm their suitability for food and nutraceutical applications.

## Figures and Tables

**Table 1:** Comparison of the degree of hydrolysis (DH%) achieved using different enzyme sources and marine substrates.

Enzyme Source	Substrate	Conditions (pH, °C, Time)	DH	References
Endogenous peptidase of viscera *T. albacares*	*Thunnus albacares* bones	pH 8 40 °C 180 min	18.96%	This study
Trypsin (Commercial)	*Thunnus albacares* bones	pH 8 40 °C 180 min	43.94%	This study
Endogenous peptidase	*Auxis rochei* viscera	Natural pH 30 °C N/A	9.0%	[43]
Endogenous peptidase	*Perna viridis*	N/A 50 °C 5 h	15.84%	[44]
Endogenous peptidase	*Chlorurus sordidus*	pH 9.0 N/A 24 h	30.65%	[45]
Endogenous peptidase	*Oncorhynchus mykiss viscera*	pH 8.0 40 °C 7 h	68.8%	[46]
Please add an explanation for the use of N/A in the table footer. Endogenous peptidase	*Oncorhynchus mykiss viscera*	pH 4 N/A 168 h	75.8%	[42]

N/A: Not reported in the cited reference.

**Table 2:** Bioactive properties (antioxidant, ACE, DPP-IV) of hydrolysates obtained with endogenous enzymes and trypsin.

Assay	Unit	Hydrolysate with Endogenous Enzymes	Hydrolysate with Trypsin
ABTS	mg Trolox/g	10.1 ± 0.12 ^b^	11.8 ± 0.04 ^a^
DPPH	mg Trolox/g	7.3 ± 0.21 ^b^	13.3 ± 0.40 ^a^
ACE	IC50 (mg/mL)	0.008 ± 0.004 ^a^	0.01 ± 0.007 ^a^
DPP-IV	IC50 (mg/mL)	1.60 ± 0.47 ^b^	0.83 ± 0.37 ^a^

Note: Values are expressed as mean ± standard deviation *(n* = 3). Different superscript letters (a, b) within the same row indicate statistically significant differences between treatments according to Student’s *t*-test (*p* < 0.05).

## Data Availability

The original contributions presented in the study are included in the article; further inquiries can be directed to the corresponding author.
